# Cuproptosis-related lncRNA signatures: Predicting prognosis and evaluating the tumor immune microenvironment in lung adenocarcinoma

**DOI:** 10.3389/fonc.2022.1088931

**Published:** 2023-01-17

**Authors:** Pengpeng Zhang, Shengbin Pei, Jianlan Liu, Xiao Zhang, Yanlong Feng, Zeitian Gong, Tianyu Zeng, Jun Li, Wei Wang

**Affiliations:** ^1^ Department of Thoracic Surgery, The First Affiliated Hospital of Nanjing Medical University, Nanjing, China; ^2^ Department of Breast Surgery, The First Affiliated Hospital of Nanjing Medical University, Nanjing, China; ^3^ Department of Burns and Plastic Surgery, The First Affiliated Hospital of Nanjing Medical University, Nanjing, China; ^4^ Department of Oncology, The First Affiliated Hospital of Nanjing Medical University, Nanjing, China

**Keywords:** lung adenocarcinoma, cuproptosis, lncRNA, prognosis, tumor immune microenvironment

## Abstract

**Background:**

Cuproptosis, a unique kind of cell death, has implications for cancer therapy, particularly lung adenocarcinoma (LUAD). Long non-coding RNAs (lncRNAs) have been demonstrated to influence cancer cell activity by binding to a wide variety of targets, including DNA, RNA, and proteins.

**Methods:**

Cuproptosis-related lncRNAs (CRlncRNAs) were utilized to build a risk model that classified patients into high-and low-risk groups. Based on the CRlncRNAs in the model, Consensus clustering analysis was used to classify LUAD patients into different subtypes. Next, we explored the differences in overall survival (OS), the tumor immune microenvironment (TIME), and the mutation landscape between different risk groups and molecular subtypes. Finally, the functions of LINC00592 were verified through *in vitro* experiments.

**Results:**

Patients in various risk categories and molecular subtypes showed statistically significant variations in terms of OS, immune cell infiltration, pathway activity, and mutation patterns. Cell experiments revealed that LINC00592 knockdown significantly reduced LUAD cell proliferation, invasion, and migration ability.

**Conclusion:**

The development of a trustworthy prediction model based on CRlncRNAs may significantly aid in the assessment of patient prognosis, molecular features, and therapeutic modalities and may eventually be used in clinical applications.

## Introduction

Lung cancer is one of the most diagnosed cancers and is the biggest cause of cancer deaths (1). The histological subtypes of lung cancer comprise small and non-small cell lung cancers (NSCLC). The latter takes up 85% of cases (2, 3). Among NSCLC, lung squamous cell carcinoma (LUSC) and LUAD represent 30% and 70% of total cases, respectively (4). LUAD is related to broad molecular heterogeneity and marked genomic changes in comparison to other lung cancer subtypes (5). Despite recent advances in immunotherapy, radiation therapy, chemotherapy, and surgery, the five-year survival rate for lung cancer patients remains extremely poor (6). Therefore, identifying a novel prognostic marker is critical for patients with LUAD.

It is widely known that during the development of multicellular organisms, there are a variety of predetermined and precisely controlled programmed cell deaths, such as necroptosis, apoptosis, pyroptosis, and ferroptosis. These forms of cell death are involved in tumorigenesis and cancer development (7–9). Cuproptosis is a novel cell death whose mechanism differs from that of known cell deaths. Researchers have found that when the known cell death mode is blocked, copper ions can still induce cell death. Copper-dependent death occurs through the direct combination of copper with the fatty acylation component of the tricarboxylic acid (TCA) cycles. This results in fatty acylation protein clustering and then iron-sulfur clustering protein loss, causing proteotoxic stress and finally cell death (10). Some investigations identified higher levels of copper in various malignancies compared to normal tissues (11). Copper aggregation is related to the proliferation and development of cancer cells, as well as the processes of angiogenesis and metastasis. In particular, higher levels of copper have been observed in the serum and tumor tissues of patients with breast, thyroid, lung, prostate, and oral cancers (12–15). There is evidence that copper may play a role in the etiology, severity, and cancer progression (16). Abnormal copper metabolism is related to many diseases, especially cancer (17–19). Since Cu metabolism holds the key to tumorigenesis, various Cu chelators, such as elesclomol, have been considered for cancer treatment. According to research reports, copper ionophores employ high amounts of copper in tumor tissues or use the susceptibility of cancer cells to oxidative stress to fight cancer (19). Targeted therapy involving copper ionophores could represent a new method of cancer treatment. As discussed in the review by Steinbrueck (20), copper-binding compounds have great potential for cancer therapy. Many different types of copper ionophores have been adopted as anticancer agents for promoting copper poisoning, including flavonoids, 8-hydroxyquinolines (HQs), bis thiosemicarbazone ligands and dithiocarbamates, among others (21). Thus, targeted copper poisoning has significant potential as a new form of cancer treatment.

Previous studies have shown that genomic variants influence tumor progression in lung cancer (22). LncRNAs are non-coding RNAs featuring transcripts of over 200 nucleotides. There has been increasing evidence to confirm that lncRNAs are involved in the progression and metastasis of NSCLC and that this is related to immune pathways. C5orf64 is exhibited as a potential index of the tumor microenvironment (TME) regulation and tumor mutation feature remodeling in LUAD (23). LINC01748 plays a carcinogenic role in non−small cell lung cancer cell lines through the regulation of the microRNA−520a−5p/HMGA1 axis (24). SLC9A3-AS1 is upregulated in NSCLC, and the SLC9A3-AS1 gene knockout hinders NSCLC progression by targeting miR-760, implying that it may have a role as a new biomarker and therapeutic target of NSCLC (25). Due to the important role of lncRNAs in cancers, the implications of lncRNAs for the prognoses of LUAD patients have been widely studied (8). However, there is an absence of research on CRlncRNAs in the prognosis and the TME of LUAD. Therefore, we employed CRlncRNAs to build a predictive model and found substantial changes in survival, pathway activity, and the TME amongst patient subgroups. Finally, cell experiments confirmed LINC00592’s effects on LUAD cells.

## Materials and methods

### Dataset acquisition and processing

The RNA sequencing data, mutation data, and corresponding clinical information of 551 LUAD patients were downloaded from the TCGA database(https://portal.gdc.cancer.gov/), which consisted of 497 LUAD tumor specimens and 54 normal specimens. Then, each gene featuring zero values was removed, and the average excess gene expression was calculated. Samples featuring no survival information and those with OS below 30 days were removed from the clinical information. The gene expression information of 397 LUAD tumor specimens and the relevant clinical information were obtained from the GEO (https://www.ncbi.nlm.nih.gov/geo/) GSE31210/GSE30219/GSE37745 datasets. For better dataset comparability, all of the expression data were converted to FPKM format. Batch effects were then eliminated using the combat function of the “sva” package. All data were transformed using Log2 before analysis. Ten cuproptosis-related genes (CRGs) were then retrieved from one published work and the researchers identified seven positively regulated and three negatively regulated CRGs (10).

### Identification of differentially expressed CRlncRNAs

The correlations between the 10 CRGs and lncRNA expression were analyzed using Pearson correlation analysis. Each CRlncRNA must be in accordance with the following standard of correlation coefficients (|Pearson R|): >0.3 and *P <*0.01. Using difference analysis, we ultimately obtained 76 differentially expressed CRlncRNAs (log2 fold alteration >1, false discovery rate (FDR) <0.05).

### Building and evaluating prognostic model

Using the “caret” R package, the 497 TCGA LUAD samples were randomly divided into a training group and a testing group at a ratio of 1: 1. The training group was used for constructing the CRlncRNAs signature, while the testing group was used for internal validation. In combination with the LUAD clinical information in TCGA, the differentially expressed CRlncRNAs were imported into the Cox and LASSO regression to create the predictive signature. Finally, the risk score was determined as follows: risk score = $\sum_{k=1}^{n}\text{Coef}\left(\text{k}\right)\;\times \text{ Expr}\left(\text{k}\right)$ . Coef (k) were the short name for the regression coefficients and Expr (k) was the expression of prognostic CRlncRNAs. Patients were divided into low- and high-risk groups based on the mean risk score. The Kaplan–Meier estimator and the log-rank test were used to analyze whether a difference existed between the high and low-risk groups in terms of the OS of LUAD patients and the “survival” R package was employed for this. Then, external validation was performed for this signature, with the risk scores of 397 LUAD samples from the GEO database calculated by the above formula for survival analysis. K-M survival curves with log-rank tests were used to assess the prognostic efficacy of the risk model across all cohorts. The accuracy of this signature in foretelling OS of LUAD patients was evaluated using receiver operating characteristic (ROC) curve analysis. Both univariate and multivariate Cox regression analyses were used to estimate the independent prognostic value.

### Nomogram and subgroups analysis of clinical features

Using the “rms” R package, the risk scores and the clinical variables of gender, age, and tumor phase were combined to construct a nomogram for the one-, three- and five-year OS of LUAD patients and correction plots based on the Hosmer-Lemeshow test for illustrating the consistency between actual outcomes and predicted outcomes. Then, we separated the clinicopathological characteristics into subgroups and ran a survival analysis to determine whether they had any effect on the model.

### Enrichment analysis

Utilizing the “GSVA” package, Gene Set Variation Analysis (GSVA) was carried out to investigate the heterogeneity of diverse biological processes. Hallmark gene sets “h.all.v7.5.1.symbols.gmt” from MSigDB (https://www.gseamsigdb.org/gsea/msigdb/index.jsp) were utilized for the GSVA. Using the R package “clusterProfiler” and “org.Hs.eg.db”, gene set enrichment analysis (GSEA) was applied to identify clearly enriched signaling paths in various groups on the basis of the following criterion: FDR < 0.25 and *P* < 0.05.

### Evaluation of the tumor immune microenvironment

In order to assess the correlation between the prognostic signature and the TIME, the immune infiltrating values of the TCGA-LUAD specimens were calculated according to 7 algorithms: CIBERSORT, CIBERSORT-ABS, EPIC, MCPCOUNTER, QUANTISEQ, TIMER, and XCELL (26–32). Spearman correlation analysis was used to evaluate the correlation between immune cell sub-populations and risk scores and the outcomes were presented in a bubble chart. Then, the abundance of immune cells and stromal cells between various groups was explored. The immune scores, stromal scores, and estimate scores (stromal scores + immune scores) of patients were computed to assess the TME differences by the “estimate” R package. Machine learning can accurately assess and quantify immunogenicity. The Cancer Immunome Atlas (TCIA) database was used to retrieve the Immunophenoscores (IPS) for LUAD (33). To forecast immunotherapy sensitivity, the IPS between the high-risk and low-risk groups was examined. Tumor Immune Dysfunction and Exclusion (TIDE) algorithm was used online (http://tide.dfci.harvard.edu/) (34). Patients were more likely to respond to ICI treatment with greater effects if their TIDE scores were lower. Then, single-sample GSEA (ssGSEA) was applied to explore the difference between various groups in terms of immune cells and immune-related function. Subsequently, comparisons were also made between high-and low-risk groups in terms of immune checkpoints.

### Drug sensitivity

The IC50 of commonly used chemotherapeutic medications in the TCGA-LUAD dataset was computed using the R package “pRRophetic” to analyze the risk model in the clinic for LUAD treatment (35). By building statistical models using gene expression and drug sensitivity data from cell lines in the Cancer Genome Project, this program enables users to predict the clinical chemotherapeutic response using just baseline tumor gene expression data. Violin plots depict the Wilcoxon signed-rank test findings comparing the IC50s of commonly used antitumor medicines between the high-and low-risk groups.

### Consensus clustering analysis, immunotherapy response, and mutations landscape

To explore potential molecular subtypes, all patients with LUAD were classified into different clusters based on the expression of prognostic CRlncRNAs *via* the R package “ConsensusClusterPlus”. Differences in survival, TIME, immune checkpoints, and response to immunotherapy between subgroups were assessed in the same manner as before. At the same time, we used the TCGA-LUAD mutation data to explore the mutation differences between high-and low-risk groups and different clusters, which were presented in the form of heat maps.

### Cell lines culture

Normal human lung epithelial BEAS-2B cells and human LUAD cell lines (A549, H1299) were purchased from the Cell Resource Center of Shanghai Life Sciences Institute. These cells were grown in F12K or RPMI-1640 (Gibco BRL, USA) with 10% fetal bovine serum (FBS), 1% streptomycin, and penicillin (Gibco, Invitrogen, Waltham, MA, USA). 5% CO2, 95% humidity, and 37°C were used to cultivate the cells.

### Cell transfection

LINC00592 knockdown was generated using small interfering RNAs (siRNAs) constructs (GenePharma, Suzhou, China). In addition, LINC00592 siRNA sequences were listed in **Supplementary Table S1**. Briefly, cells were seeded at 50% confluence in a 6-well plate and infected with negative control (NC), and knockdown (siLINC00592). All transfections were carried out with Lipofectamine 3000 (Invitrogen, USA).

### Extraction of RNA and real-time PCR

Total RNA from cell lines was extracted by the manufacturer’s instructions using TRIzol (15596018, Thermo). After that, cDNA was created using the PrimeScriptTMRT kit (R232-01, Vazyme). SYBR Green Master Mix (Q111-02, Vazyme) was used to perform the Real-time polymerase chain reaction (RT-PCR), and the expression levels of each mRNA were normalized to the level of mRNA GAPDH. The 2^−ΔΔCt^ method was used to count the expression levels. Tsingke Biotech (Beijing, China) provided all primers, and **Supplementary Table S1** has full primer sequences.

### Cell counting kit-8 experiment

In 96-well plates, we seeded the cell suspension with 3×10^3^ cells per well. The plate was then incubated for 2 hours at 37°C in the dark with 10 mL of CCK-8 labeling agent (A311-01, Vazyme) each well. The enzyme-labeled meter (A33978, Thermo) was used to measure the absorbance of the cells at 450 nm for 0, 24, 48, 72, and 96 hours in order to determine the vitality of the cells.

### Colony formation

We transfected 1×10^3^ cells into each well of a 6-well plate and kept the cells alive for 14 days. Before Crystal violet (Solarbio, China) staining, the cells were washed twice with PBS and fixed for 15 minutes in 4% paraformaldehyde.

### EdU

A 96-well plate with 2×10^4^ treated cells in each well was used for the experiment, which was conducted after the cells had attached to the wall. The manufacturer’s recommended 5-Ethynyl-2’-deoxyuridine (EdU) assay was then carried out (Ribobio, China). An inverted microscope was used to count the number of proliferating cells.

### Wound-healing assay

In 6-well plates, transfected cells were plated and cultured in a cell incubator until they were 95% confluent. In each cultured well, a single straight line was scraped using a sterile 20-L plastic pipette tip, and unattached cells and debris were gently washed away twice with PBS. Finally, we used the Image J software to measure the width of the scratches after taking photos of the scratch wounds at 0h and 48h.

### Transwell assay

Cell invasion and migration studies were performed in transwells. The top chamber of 24-wells was filled with treated A549 and H1299 cells (2×10^5^), which were then incubated for 48 hours. To assess the cells’ ability to invade and migrate, the top section of the plate was either precoated with matrigel solution (BD Biosciences, USA) or left untreated. The remaining cells on the bottom layer were then fixed with 4% paraformaldehyde and stained with 0.1% crystal violet after the cells on the top surface were removed (Solarbio, China).

### Statistical analysis

All of our data and statistics were processed using R (version 4.1.3). Experiment data were analyzed using the applications Graphpad and Image J. Kaplan-Meier curves with a log-rank test were used to compare the OS rates of the two groups. A “Survminer” R package was used to generate all survival curves. To assess prognostic factors, univariate and multivariate Cox regression analysis (CRA) was employed. Lasso regression was used to discover characteristics that had a greater impact on the outcomes. The “ggplot2” R program was used to visualize the data, and the “survival” R package was used to calculate the OS and risk ratings. The heatmap is created using the “Pheatmap” R package. For normally distributed data, a two-tailed t-test or a one-way ANOVA was employed to discover significant quantitative differences. To determine if there were significant differences for nonnormally distributed data, a Wilcoxon test or a Kruskal-Wallis test was utilized. Every statistical analysis was completed using R software. *P* < 0.05 is a statistically significant value.

## Outcomes

### Conformation of CRlncRNAs

**Figure 1:**
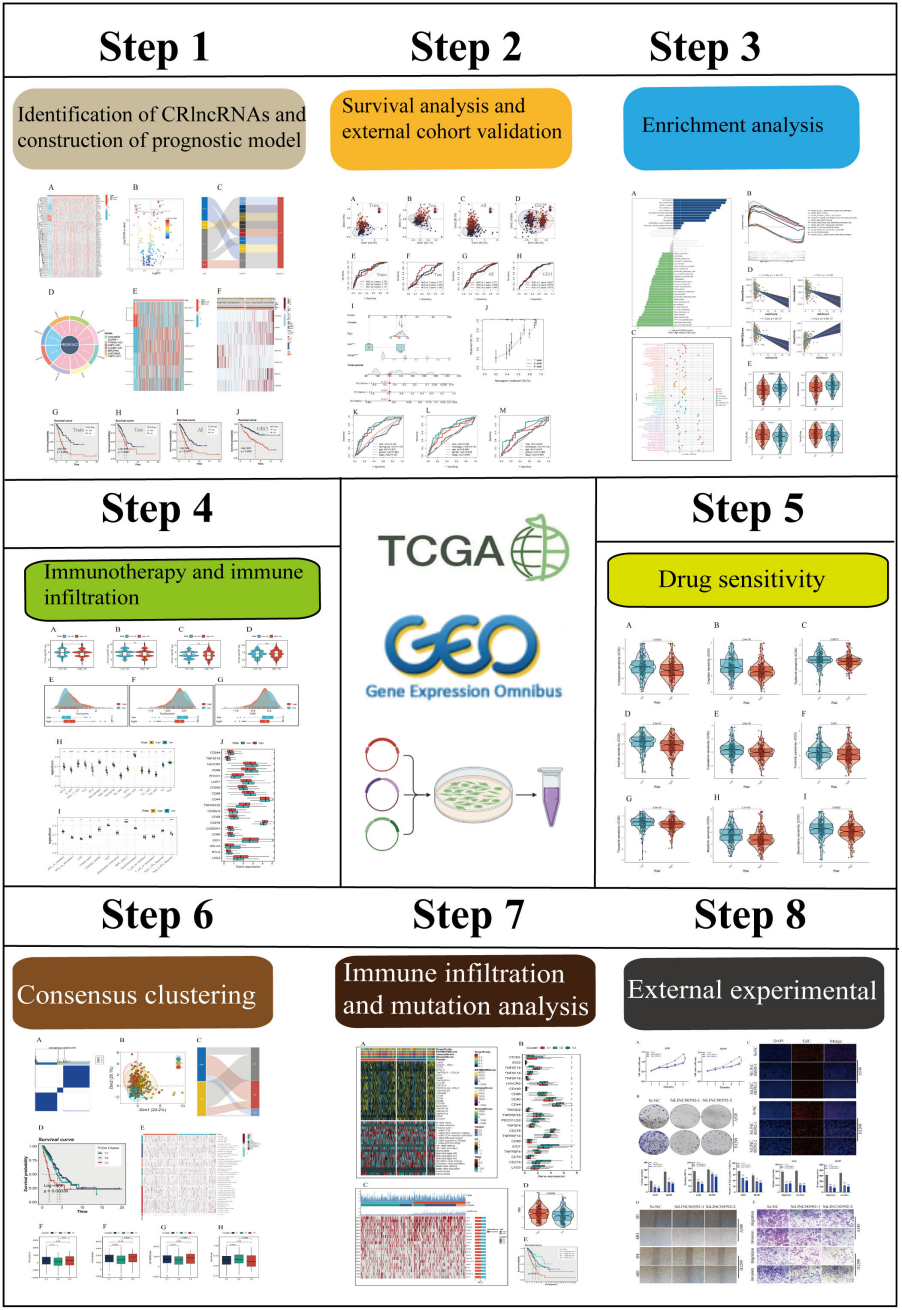
The flowchart of this study.

**Figure 2:**
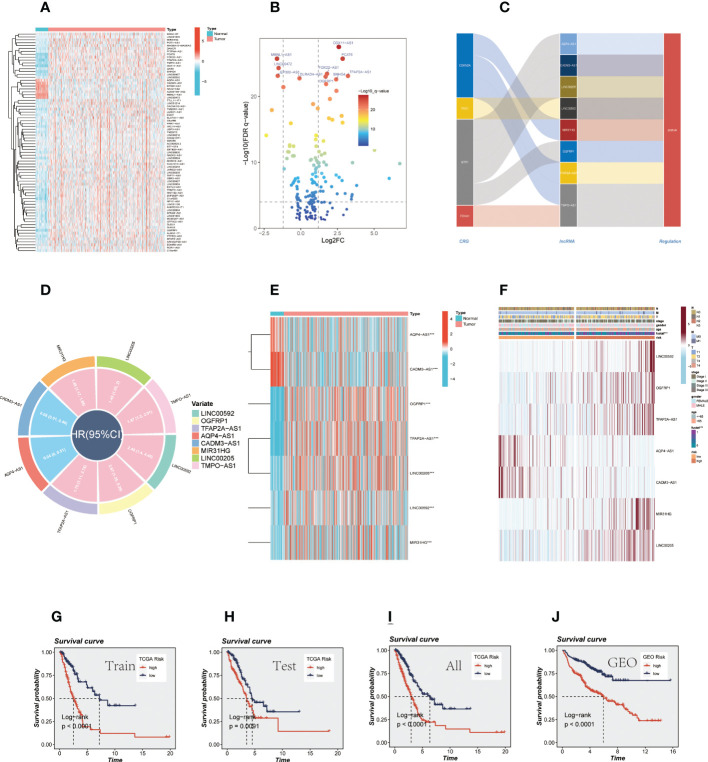
CRlncRNAs prognostic signature in LUAD. **(A)** A heat map depicted differentially expressed CRlncRNAs between tumor and normal samples and the top ten genes are annotated. **(B)** A volcano diagram depicted differentially expressed CRlncRNAs. **(C)** The sankey diagram shows the regulatory relationship between CRGs and 7 prognostic CRlncRNAs. **(D)** Circle diagram shows the HR value and confidence interval of 7 prognostic CRlncRNAs. **(E)** Difference between normal and tumor tissue of 7 prognostic CRlncRNA. **(F)** A heatmap of correlations between prognostic signatures of CRlncRNAs and clinicopathological outcomes. **(G-J)** Survival analysis for TCGA cohort (training group, testing group, and all group) and GEO cohort.

The flow chart of this study was shown in **Figure 1**. A total of 76 differentially expressed CRlncRNAs were obtained by correlation analysis (|Pearson R| > 0.3 and *P* < 0.01) and variance analysis (|Log2FC| > 1 and *P* < 0.05), and the correlation network diagram (**Supplementary Figure S1A**) and corresponding heatmap and volcano charts were constructed (**Figures 2A, B**).

### Construction and validation of the CRlncRNAs signature

Through univariate CRA, 8 prognostic CRlncRNAs related to OS (*P* < 0.05) were obtained, and each of the CRlncRNAs was modulated positively through CRGs as shown in the Sankey diagram (**Figure 2C** **).** The circle diagram displays the prognostic HR value of 8 CRlncRNAs, and it is clear that TPMO-AS1, LINC00205, OGFRP1, MIR31HG, LINC00592, OGFRP1, and TFAP2A-AS1 are high-risk genes. In contrast, AQP4-AS1 and CADM3-AS1 were shown to be low-risk genes (**Figure 2D**). Subsequently, the prognostic signature consisting of 7 CRlncRNAs (LINC00592, OGFRP1, TFAP2A-AS1, AQP4-AS1, CADM3-AS1, MIR31HG, and LINC00205) was constructed by LASSO regression (**Supplementary Figures S1B, C**) and multivariate CRA. Some of the prognostic CRlncRNAs (LINC00205, OGFRP1, MIR31HG) have previously been proven to be closely related to NSCLC (36–38). Then, the expression levels of these genes were examined in the normal and tumor groups (**Figure 2E**), and the findings revealed that AQP4-AS1 and CADM3-AS1 were strongly expressed in the normal group, while the other genes were substantially expressed in the tumor group. Meanwhile, the clinical correlation heatmap was constructed based on clinical information and the expression levels of the 7 CRlncRNAs (**Figure 2F**). The risk scores of all LUAD patients were then calculated according to regression coefficients, and the patients were divided into high-and low-risk groups based on the mean risk score. The risk scores were calculated using the following formula: risk score = (0.6418 × LINC00592 expression) + (0.6236 × OGFRP1 expression) + (0.5246 × TFAP2A-AS1 expression) + (-2.4203 × AQP4-AS expression) + (-1.47395306825907 × CADM3-AS1 expression) + (0.3009 × MIR31HG expression) + (0.5019 × LINC00205 expression). Similarly, we calculated the risk value for 397 LUAD samples from the GEO database using the above formula and subsequently divided them into high-and low-risk groups. In order to determine the prognostic capability of the risk signature, we compared Kaplan-Meier survival plots for OS (**Figures 2G-J**), the distribution of risk scores, the survival time and survival status, and the related expression of 7 CRlncRNAs between the high-and low-risk groups in the TCGA cohort (training group, the testing group, all groups) and the GEO cohort (GSE31210, GSE3021,9 and GSE37745) (**Supplementary Figures S1D-G**).

### Evaluation of prognostic models and nomograms

**Figure 3:**
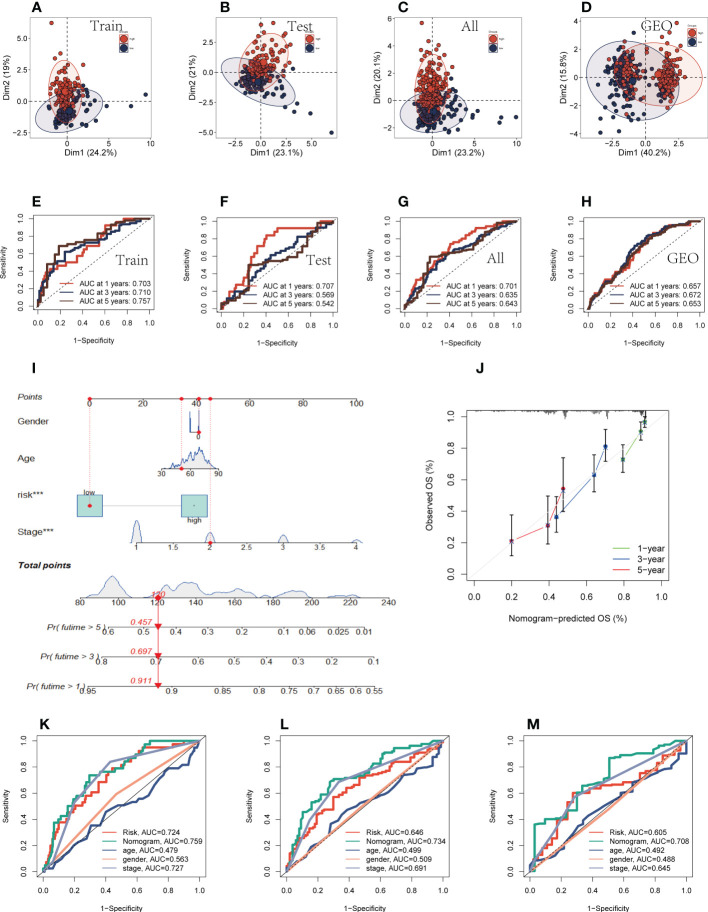
Evaluation of prognostic models and nomograms. **(A–D)** PCA analysis in TCGA cohort and GEO cohort. **(E–H)** Time-dependent ROC curves of signatures in the TCGA cohort (training group, testing group, and all group) and GEO cohort. **(I)** Nomogram was constructed by combining Clinical features with risk groups. **(J)** Nomogram’s 1-, 3, and 5-years calibration curve. **(K–M)** ROC curves for 1, 3, and 5 years showed AUC values for various clinical factors, risk scores, and nomogram scores.

**Figure 4:**
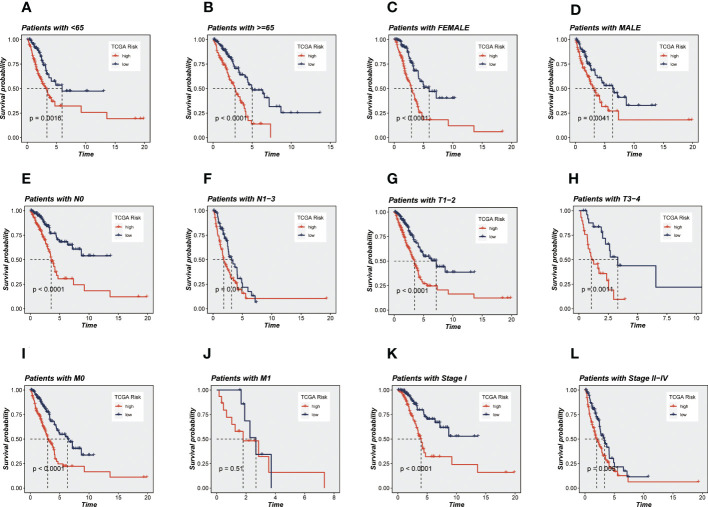
KM survival analyses of various clinical subgroups in TCGA-LUAD cohort. **(A)** Age ≤ 65 years; **(B)** Age > 65 years; **(C)** Female; **(D)** Male; **(E)** Pathology N0; **(F)** Pathology N1-3; **(G)** Pathology T1-2; **(H)** Pathology T3-4; **(I)** Pathology M0; **(J)** Pathology M1; **(K)** Stage I; **(L)** Stage II-IV. It can be seen that the ability of the model to predict survival was not affected by clinical subgroups.

Univariate and multivariate CRA confirmed that our risk model was an independent prognostic indicator in TCGA and GEO cohorts (*P <*0.001, **Supplementary Figures S1H-K**). After this, it can be demonstrated using principal component analysis (PCA) analysis that the risk score may separate the TCGA and GEO cohorts into two different clusters (**Figures 3A-D**). Then, the ROC curves were used for validation of the specificity and sensitivity of the signatures in train groups, and the AUCs for one-, three- and five-year survival reached 0.703, 0.710, and 0.757, respectively, implying a good predictive value (**Figure 3E**). Additionally, the ROC curves showed high predictive abilities in the other validation groups (**Figures 3F-H**). After that, we evaluated the ability of the model genes to predict the high-and low-risk of patients, and the results are shown in **Supplementary Figures S2A-G**. The AUC values were all greater than 0.6, among which the AUC values of OGFRP1 and LINC00205 were both greater than 0.7, showing a strong predictive ability. Clinical data and risk classification were merged to construct a nomogram (**Figure 3I**), which was then utilized to evaluate the prognosis of TCGA-LUAD patients. The nomogram may aid in more precisely determining patient risk and directing future treatment options. We also created calibration curves (**Figure 3J**) and observed that this nomogram could reliably predict the prognosis of LUAD patients after one-, three-, and five years. Prognostic ROC analysis was used to further evaluate the accuracy of this nomogram, and the findings significantly beat those of other clinical shapes and risk scores. The AUC in 1, 3, and 5 years was 0.724, 0.734, and 0.708, respectively, according to the results (**Figures 3K-M**). These outcomes further suggested that this predictive signature constitutes a promising biomarker to predict the prognosis of LUAD. To determine whether the constructed prognostic model could forecast OS based on various clinical features, we conducted survival analysis for several clinical subgroups. The following analytical details are provided: Age (≤65 or >65), Gender (Male or Female), Clinical Stage (Stage I or Stage II-IV), T Status (T1-2 or T3-4), M Status (M0 or M1), and N Status (N0 or N1-3). **Figures 4A-L** showed that independent of age, gender, clinical stage, T status, M status, or N status, the OS rates of high-risk patients were lower than those of low-risk patients.

### Analysis of enrichment

**Figure 5:**
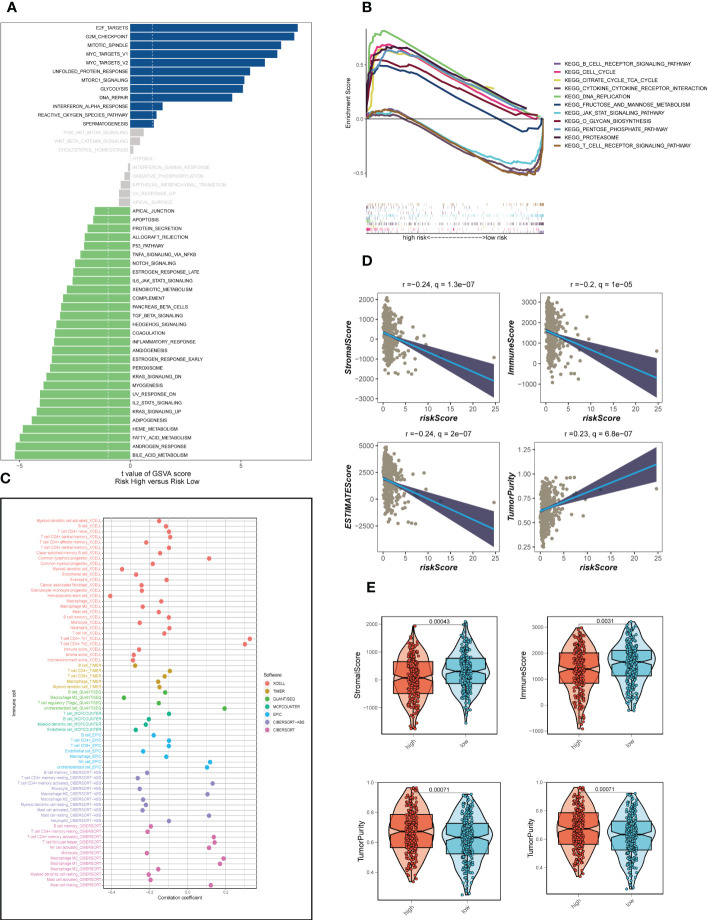
Enrichment analysis and assessment of immune infiltration. **(A)** GSVA showed the enrichment of hallmark gene sets in different risk groups. **(B)** GSEA enrichment method showed pathway differences between high-and low-risk groups. **(C)** The correlation between risk scores and tumor-infiltrating immune cells. **(D)** A scatter plot depicted the correlation of risk scores with stromal score, immune score, and ESTIMATE score. **(E)** The violin charts the differences of stromal score, immune score, and ESTIMATE score, respectively, between low-and high-risk groups.

Analysis of hallmark pathway gene signatures highlighted that the high-and low-risk groups showed some differences. A direct comparison of Risk-High versus Risk-Low revealed that the top 5 enriched signatures in high-risk group included E2F targets, G2M checkpoint, MYC targets v1, MYC targets v2 and unfolded protein response and the low-risk group was mainly active in some metabolic related pathways (**Figure 5A**). According to clinical research, E2F family members are directly linked to the incidence, growth, proliferating, and apoptosis of cancerous tumors such as gastric, pulmonary, liver, esophagus, prostrate, bladder, and ovarian cancer (36, 37). In order to control cell proliferation, the G2M checkpoint also functions as a cell cycle regulatory route. High G2M checkpoint pathway activation has been linked in studies to considerably worse survival in people with pancreatic cancer (38). Furthermore, c-Myc is necessary for tumorigenesis (39), Almost often, Myc may increase transcription (40), which showed that LUAD cells could be susceptible to Myc inhibition. Extremely crucial nuclear transcription factors involved in controlling the cell cycle are encoded by the E2F family (36, 41). Unfolded protein response (UPRmt) is known to be preserved in cancer and is able to become active in response to mitochondrial stress (42). This helps to preserve mitochondrial integrity and promotes the development of tumors. As a result, these pathways, which were more prevalent in the high-risk group, may play a crucial role in controlling tumor development in LUAD. Then, GSEA was further applied to determine the obvious pathways between different risk groups (**Figure 5B**). It was found that the T/B cell receptor signaling pathway (SP), the JAK/STAT SP, and the cytokine receptor interaction were clearly enriched in the low-risk groups, implying a close association of low-risk patients with tumor- and immune-related pathways. On the other hand, high-risk groups were obviously enriched in cell cycle, DNA replication and proteasome pathway.

### Assessment of TIME

**Figure 6:**
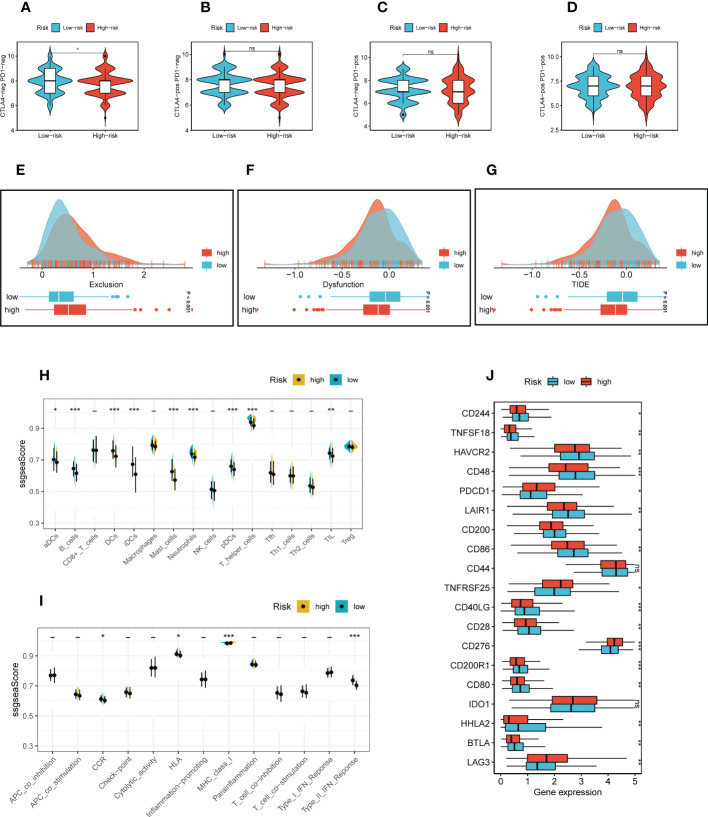
Immunotherapy and immune checkpoint analysis. **(A-D)** The low-risk group showed higher IPS scores when patients were not treated with PD1/PDL1 and CTLA-4, and no significant differences were seen in the other subgroups. **(E-G)** TIDE scores were significantly lower and Exclusion scores were significantly higher in the high-risk group than in the low-risk group. **(H, I)** The ssGSEA scores of immune cells and immune functions in two risk groups. **(J)** The difference of common immune checkpoint expression in different risk groups. ***P ≤ 0.001. **P ≤ 0.01. *P ≤ 0.05.

The correlation between risk scores and tumor-infiltrating immune cells was explored (**Figure 5C**). Most of the levels of immune cell infiltration, such as memory activated CD4+T cells, B cells and macrophages, were negatively correlated with the risk score, which may imply that patients in the low-risk group had higher levels of immune cell infiltration in the TME, as we confirmed in the following validation. In order to further assess TIME patterns and immunotherapeutic responses across different risk groups, we conducted an attempt. The components of LUAD’s TME score were calculated using the ESTIMATE method. Correlation analysis showed that the risk score was negatively correlated with immune scores, stromal cores and estimate scores, and positively correlated with tumor purity (**Figure 5D**). The violin diagrams confirmed that the low-risk group had higher immune scores, stromal scores and estimate scores, and the high-risk group had higher tumor purity (**Figure 5E**). The IPS may also aid in identifying those who might benefit from immunotherapy. It was predicted that LUAD patients will respond well to either PD-1/PD-L1 or CTLA4 inhibitors, or perhaps to both (**Figures 6A-D**). The results showed that the low-risk group showed higher IPS scores when patients were not treated with PD1/PDL1 and CTLA-4, and no significant differences were seen in the other subgroups. Surprisingly, Dysfunction scores were much lower in the high-risk group and Exclusion scores were significantly greater than in the low-risk group (*P* < 0.001; **Figures 6E-G**), which suggests that high-risk patients are more likely to benefit from immunotherapy. Subsequently, the ssGSEA enrichment scores of different immune cells and immune related functions were quantified (**Figures 6H, I**). Compared to the high-risk group, B cells, DCs, immature dendritic cells (iDCs), mast cells, neutrophils, plasmacytoid dendritic cells (pDCs) and T helper cells had higher enrichment scores in the low-risk group. In the low-risk group, CCR, HLA, MHC class I and Type II TFN response were enriched to a higher degree, which may indicate that the TME of low-risk patients has a more active immune status to fight against tumor progression and thus has a better prognosis. Meanwhile, a comparison of various risk groups in terms of immune checkpoint activation revealed that most immune checkpoints expressed more activity in low-risk groups, such as HHLA2, CD40LG, CD48 and CD244 (**Figure 6J**). Some of these immune checkpoint expression levels were found to be highly expressed in the high-risk group, such as LAG3, IDO1, CD276 and PDCD1 (also called PD1), which may provide therapeutic guidance for high-risk groups.

### Drugs sensitivity

**Figure 7:**
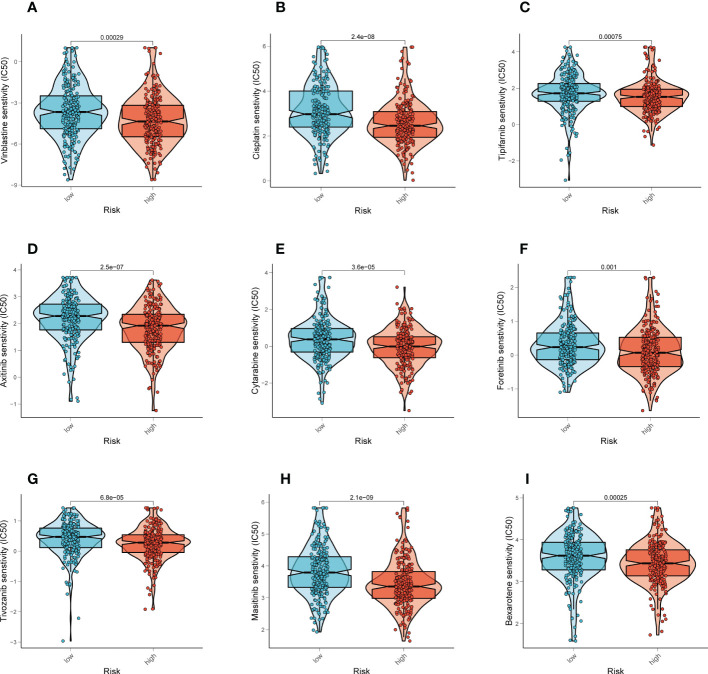
Prediction of chemotherapy drug sensitivity in LUAD patients. **(A-I)** Comparisons of IC50 for chemotherapeutics between two subgroups revealed that the high-risk group was more likely to benefit from Vinblastine, Cisplatin, Tipifarnib, etc.

Different chemotherapeutic medicines’ effectiveness was compared between different groups. For patients with higher risk score, we found that the IC50 for the following chemotherapeutic medications was lower: Vinblastine, Cisplatin, Tipifarnib, Axitinib, Cytarabine, Foretinib, Tivozanib, Masitinib, Bexarotene (*P* < 0.001, **Figures 7A-I**). Our results support the use of this risk score to forecast chemotherapeutic drug sensitivity and immunotherapy response in LUAD patients, which makes it easier to construct a personalized medication treatment.

### Consensus clustering and TME

**Figure 8:**
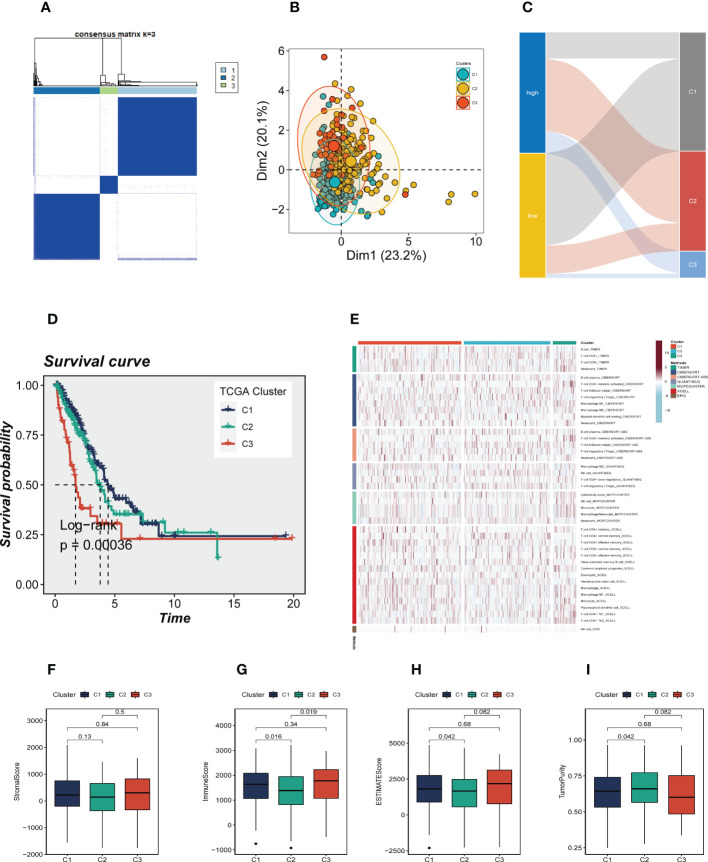
Consensus Clustering based on 7 prognostic CRlncRNAs expression. **(A)** LUAD patients were divided into three clusters (k=3). **(B)** PCA depicted the distribution for clusters. **(C)** The Sankey diagram of the connection between clusters and high-and low-risk group. **(D)** Kaplan–Meier survival curves of OS in clusters. **(E)** A heat map showing immune infiltration containing 7 algorithms in different clusters. **(F-H)** The boxplots showed that the differences of stromal score, immune score, and ESTIMATE score, respectively, in three clusters. **(I)** The boxplots showed that the differences of stromal score, immune score, ESTIMATE score, and tumor purity respectively, in three clusters.

In order to identify distinctive molecular subtypes according to the expression of prognostic CRlncRNAs, unsupervised consensus clustering was applied. LUAD patients were separated into three clusters, with k=3 identified as the optimal clustering stability (**Figure 8A**). The PCA plot showed the distribution of Cluster 1(C1), Cluster 2 (C2) and Cluster 3 (C3) samples (**Figure 8B**), and a Sankey diagram was constructed to display the connection between clusters and high-and low-risk groups (**Figure 8C**). Samples C2 and C3 mainly belong to the high-risk group, while samples C1 are mainly distributed in the low-risk group. Subsequent survival analysis showed that patients in the C3 group had the worst prognosis, while patients in the C1 group had the best prognosis (**Figure 8D**). The heat map of immune cell infiltration including 7 algorithms was drawn (**Figure 8E**), and the results showed that there was higher immune cell infiltration in the TME of C1 group, such as M2 macrophages, T cells, etc., which may mean that more immune cells in the TME of C1 were recruited and activated adaptive immune response to form a hot tumor state. The TME scores of various clusters were then analyzed, and the findings revealed that C1 had a higher immune score, stromal score and estimate score, as well as lower tumor purity, than C2.

### Immunotherapy and the mutational landscape

**Figure 9:**
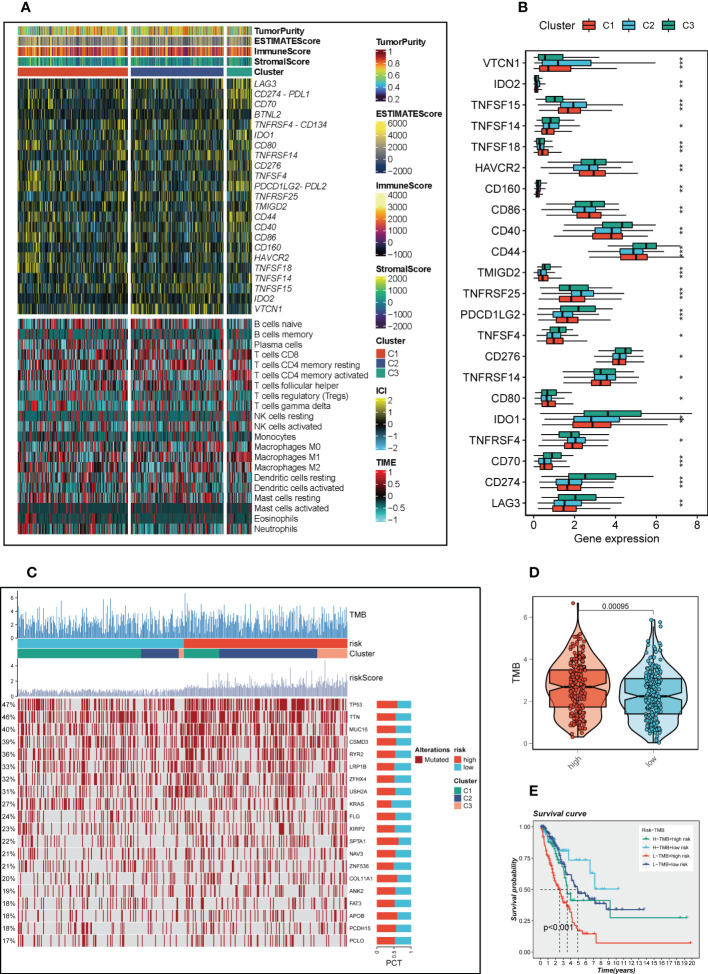
The TIME and the mutation landscape based on three clusters. **(A)** A heat map represented TME score, immune checkpoint and immune cell infiltration in three clusters **(B)** The differences of immune checkpoints expression in three clusters. **(C)** Mutational landscapes in different risk groups and clusters. **(D)** Differences in TMB between high-and low-risk groups. **(E)** Survival analysis for four groups (H-TMB+high-risk, H-TMB+low-risk, L-TMB+high-risk, and L-TMB+low-risk). *P < 0.05, **P < 0.01, ***P < 0.001.

TME scores, immune checkpoint and immune cell infiltration results calculated by CIBERSORT were integrated to draw a heat map (**Figure 9A**), which showed that there were different degrees of highly expressed immune checkpoints in the C3 group, such as LAG3, PDL1, CD70, IDO1, PDL2, etc. This may indicate that C3 group is more likely to benefit from immune checkpoint blockade treatment, whereas no significant difference was seen in the heat map of immune cell infiltration. Subsequently, the expression of immune checkpoints in different clusters is shown by boxplot (**Figure 9B**). Patients with LUAD mutational landscape were presented in **Figure 9C**, which showed the highest 20 mutation frequency of gene mutation, it can be seen in the high-risk group, C2/C3 gene mutation frequency increases. **Figure 9D** confirmed that higher tumor mutation burden in high-risk groups, so we further according to the median risk scores and the median TMB, divided the patients into four groups (H-TMB+high-risk, H-TMB+low-risk, L-TMB+high-risk, and L-TMB+low-risk) and the survival differences between them were further observed (**Figure 9E**). Survival analysis illustrated that H-TMB+low-risk group had the best prognosis and L-TMB+high-risk group had the worst prognosis.

### Validation of LINC00592 expression and biological function in LUAD

**Figure 10:**
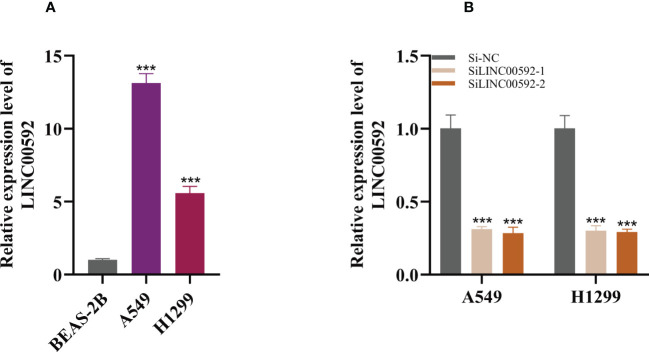
Cell experiment. **(A)** qRT-PCR to evaluate the level of LINC00592 expression in healthy human lung epithelial BEAS-2B cell lines and two LUAD (A549, H1299) cell lines **(B)** qRT-PCR to evaluate the level of LINC00592 expression 5 days after transfection and siRNA sequences could result in significant decrease in LINC00592 expression ***P < 0.001.

**Figure 11:**
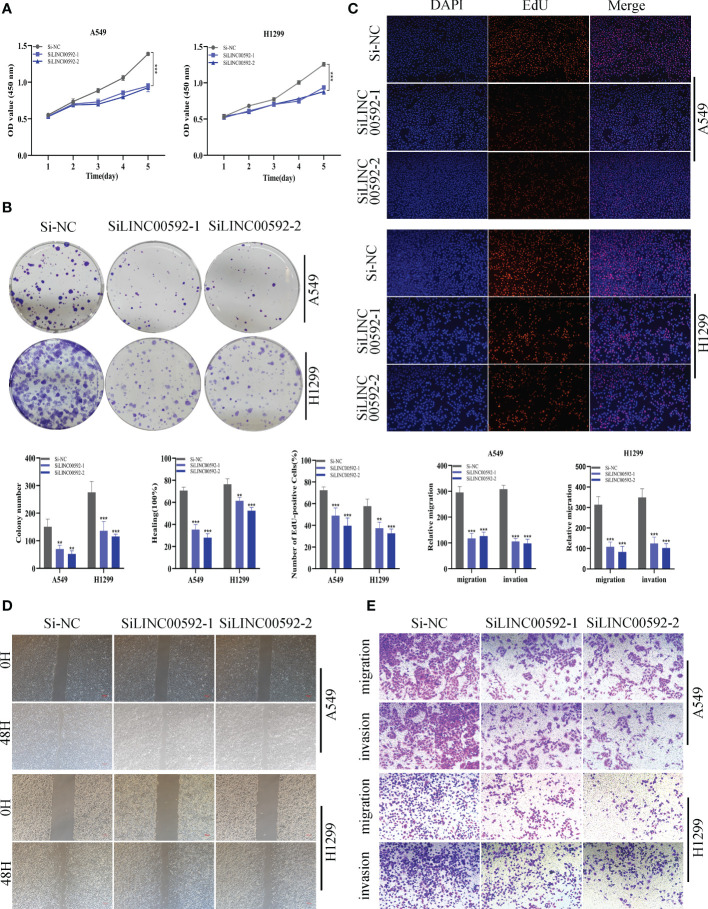
The role of LINC00592 in LUAD. **(A)** CCK8 assay showed that, after LINC00592 knockdown, the cells showed significant reduction in viability. **(B)** Colony formation assay displayed that cell with reduced LINC00592 expression exhibited a significant reduction in the numbers of colonies, compared with the NC group. **(C)** EdU staining assay indicated that downregulation of LINC00592 expression repressed cell proliferation in LUAD cell lines. **(D)** Scratch-wound healing assay depicted that a significantly slower wound healing rate was observed in cells with a decreased expression of LINC00592. **(E)** Transwell assay showed that downregulation of LINC00592 expression inhibited the migration and invasion capacity of LUAD cells. To demonstrate the accuracy and reproducibility of the results, all experiments were repeated in two LUAD (A549, H1299) cell lines and all data were presented as the means ± SD of three independent experiments. **P < 0.01, ***P < 0.001.

In the above studies, we found that LINC00592 exhibited the highest HR value and was substantially elevated in tumor samples compared to normal samples. Using the GEPIA database, we further determined the impact of LINC00592 on survival, and patients with high expression of LINC00592 had a worse prognosis (**Supplementary Figure S2H**). Further research was done on the LINC00592 in the signature. We conducted *in vitro* research to better comprehend LINC00592’s role in LUAD. Compared to BEAS-2B cell lines from healthy human lung epithelial tissue, **Figure 10A** showed that LINC00592 was significantly elevated in two LUAD (A549, H1299) cell lines. LINC00592 in LUAD cell lines was knocked down for subsequent experiments. First, we employed the qRT-PCR technique to measure the level of LINC00592 expression 5 days after transfection in order to gauge the effectiveness of siRNA knockdown of LINC00592 in A549 and H1299 cell lines (**Figure 10B**). We discovered that all siRNA sequences could significantly reduce LINC00592 expression (*P* < 0.001). Following LINC00592 knockdown, there was also a significant reduction in the cells’ vitality as evaluated by the CCK8 assay (**Figure 11A**). The findings of the experiment indicate that LINC00592 may have a key role in LUAD cell surviva. Cell proliferation was evaluated using the colony formation assay. Comparing cells with decreased LINC00592 expression to the siRNA NC group, the reduced LINC00592 expression cells showed a significantly lower number of colonies (**Figure 11B**). Therefore, a slower rate of colony formation was seen in LINC00592 knockdown cells, suggesting that LINC00592 may be essential for the proliferation of the LUAD cell line. EdU assays revealed that lower expression of LINC00592 decreased the proliferative activity of A549 and H1299 cells in comparison to the NC group (**Figure 11C**). The outcome of the scratch-wound healing experiment was comparable. Cells with reduced LINC00592 expression showed a significantly delayed rate of wound healing (**Figure 11D**). Transwell experiments revealed that LINC00592 downregulation severely restricted the migration and invasion of A549 and H1299 cells (**Figure 11E**). To demonstrate the accuracy and reproducibility of the results, all experiments were repeated in two LUAD (A549, H1299) cell lines and all data were presented as the means ± SD of three independent experiments. *P < 0.05, **P < 0.01, ***P < 0.001.

## Discussion

As the most frequently diagnosed subtype of NSCLC, LUAD still presents a great challenge to global human health, with mortality and morbidity continuing to rise. Identifying an effective and reliable prognostic signature for patients with LUAD is critical for improving its prognosis. There has been an accumulation of data demonstrating that the abnormal behaviors of lncRNAs, such as over-expression, mutation or deletion, constituted drivers for recurrence, metastasis and progression of tumors (43). A study showed that miR-142-3p acted as a tumor inhibitor in NSCLC *via* inhibition of the MALAT1/b-catenin SP (44). In conclusion, lncRNAs were involved in numerous key biological LUAD courses. Despite the discovery of numerous other lncRNA signatures for predicting LUAD survival results, a CRlncRNA signature hadn’t yet been identified. Therefore, such a signature was developed for exploring the prognoses and the TME of LUAD patients.

In the exploration, we performed COX and LASSO analysis based on the differentially expressed CRlncRNAs and finally obtained 7 CRlncRNAs (LINC00592, OGFRP1, TFAP2A-AS1, AQP4-AS1, CADM3-AS1, MIR31HG, and LINC00205) that were most relevant to the prognosis of LUAD patients. Meanwhile, we conducted both internal and external validation to evaluate the accuracy of the signature. Of these prognostic CRlncRNAs, LINC00205 was identified as promoting malignant tumors of LUAD by sponging miR-185-5p (45); OGFRP1 constitutes an oncogene in NSCLC through the miR-4640-5p/eIF5A axis, and downregulation of OGFRP1 inhibited the progression of NSCLC (46); MIR31HG could be confirmed as a poor prognostic biomarker and a new therapeutic target for NSCLC patients through activation of the Wnt/β−catenin SP (47); AQP4-AS1 is downregulated in breast cancer tissues, and its over-expression is related to better prognoses (48); however, for the first time, LINC00592, TFAP2A-AS1 and CADM3-AS1 were unveiled in lung cancer and could represent new therapeutic targets. The LUAD patients were then classified into low-and high-risk groups according to the median risk score for subsequent analysis. The results revealed a better prognosis in the low-risk group than the high-risk group, and risk scores were independent predictors of OS of LUAD patients. ROC analysis showed the signature to be relatively accurate in predicting LUAD survival. The nomogram presented an excellent degree of consistency between the observed and predicted rates for the one-, three-and five-year OS. Taken as a whole, the above results suggest that our CRlncRNAs signature may be highly accurate in predicting the prognoses of LUAD patients.

The GSVA results showed that the high-risk group was mainly enriched in cell cycle-related pathways, such as E2F targets, G2M checkpoint, and MYC targets, whose activation may promote tumor progression, while the low-risk group was mainly enriched in metabolism-related pathways, such as fatty acid metabolism and bile acid metabolism. GSEA results indicated that the JAK/STAT SP, cytokine receptor interaction, and T/B cell receptor SP were all enriched in low-risk groups. The researchers discovered that the activating JAK2 p.V617F mutation might confer sensitivity to anti-PD1 immunotherapy and JAK suppressors in NSCLC (49). The upregulation, mutation, and amplification of JAK2 detected may participate in the invasion, migration, and proliferation of cancer cells in LUAD (50). Suppression of the IL-6R/JAK1/STAT3 SP raised the degree of sensitivity to afatinib in NSCLC (51). To further evaluate the immune infiltration status of different risk groups, we used seven different algorithms to verify the higher immune cell infiltration level in the low-risk group. Following that, ssGSEA enrichment analysis also showed similar results, indicating that the low-risk group was more prone to form a hot tumor state to activate the immune system to resist tumor progression. Studies have revealed immune checkpoint gene expression levels to be closely related to the efficacy of immunotherapy (52). According to our study, some immune checkpoints were differentially expressed in both high-and low-risk groups. To further explore the effect of immunotherapy benefit in different risk groups, we explored the situation of TIDE scores in different risk groups, and the results showed that the low-risk group had higher TIDE scores, which also indicated that the high-risk group may be more suitable for immunotherapy.

Molecular subtypes have been reported to be related to the TME (53). TME status differ among subtypes, leading to differences in prognosis and response to immunotherapy. Patients were classified into three clusters according to the expression of the 7 prognostic CRlncRNAs, and the three clusters were compared in terms of survival, the TME and immune checkpoints. The results showed that C1 exhibited the highest OS. C3 featured a higher abundance of immune and stromal cells. Also, most of the immune checkpoints were highly expressed in C3, indicating that patients in C3 are more sensitive to immunotherapy than those in the other two groups. Subsequently, mutation analysis showed the differential landscape of gene mutations in different risk groups and different clusters, which showed that the high-risk group had significantly higher TMB, and further survival analysis found that H-TMB+low-risk group had the best OS rate.

According to our study, LINC00592 is highly expressed in tumor cells and has the largest HR value. Survival analysis showed that LINC00592 has a significant effect on survival, patients with high LINC00592 expression have a worse prognosis. Cell experiments confirmed that knockdown of LINC00592 reduced the proliferation, invasion and migration of LUAD cells. LINC00592 may be a new therapeutic target for LUAD patients, so it was used for experimental validation.

Inevitably, our study has some limitations and shortcomings. Our studies of the functional phenotype of LINC00592 have been preliminary, and its particular functions and mechanisms in LUAD need additional research utilizing animal models *in vivo*. In summary, the CRlncRNAs signature was capable of independently predicting LUAD patients’ prognoses, and may provide guidance for immunotherapy for LUAD patients.

## Data availability statement

The original contributions presented in the study are included in the article/**Supplementary Material**. Further inquiries can be directed to the corresponding authors.

## Author contributions

PZ, SP, JLL, and XZ contributed conception and design of the study. XZ, YF and ZG collected the data. PZ and SP performed the statistical analysis. PZ wrote the first draft of the manuscript. JL and TZ revised the manuscript. WW, JL and TZ gave the final approval of the version to be submitted. All authors contributed to manuscript and approved the submitted version.
