# Supplementary material for: Cuproptosis-related lncRNA signatures: Predicting prognosis and evaluating the tumor immune microenvironment in lung adenocarcinoma
Source: Front Oncol. 2023 Jan 17;12:1088931. doi: 10.3389/fonc.2022.1088931 (PMC9887198; doi:10.3389/fonc.2022.1088931)
Supplement: Supplementary file 1 [file Image_1.pdf]

Supplementary Material

1 Supplementary Figure S1

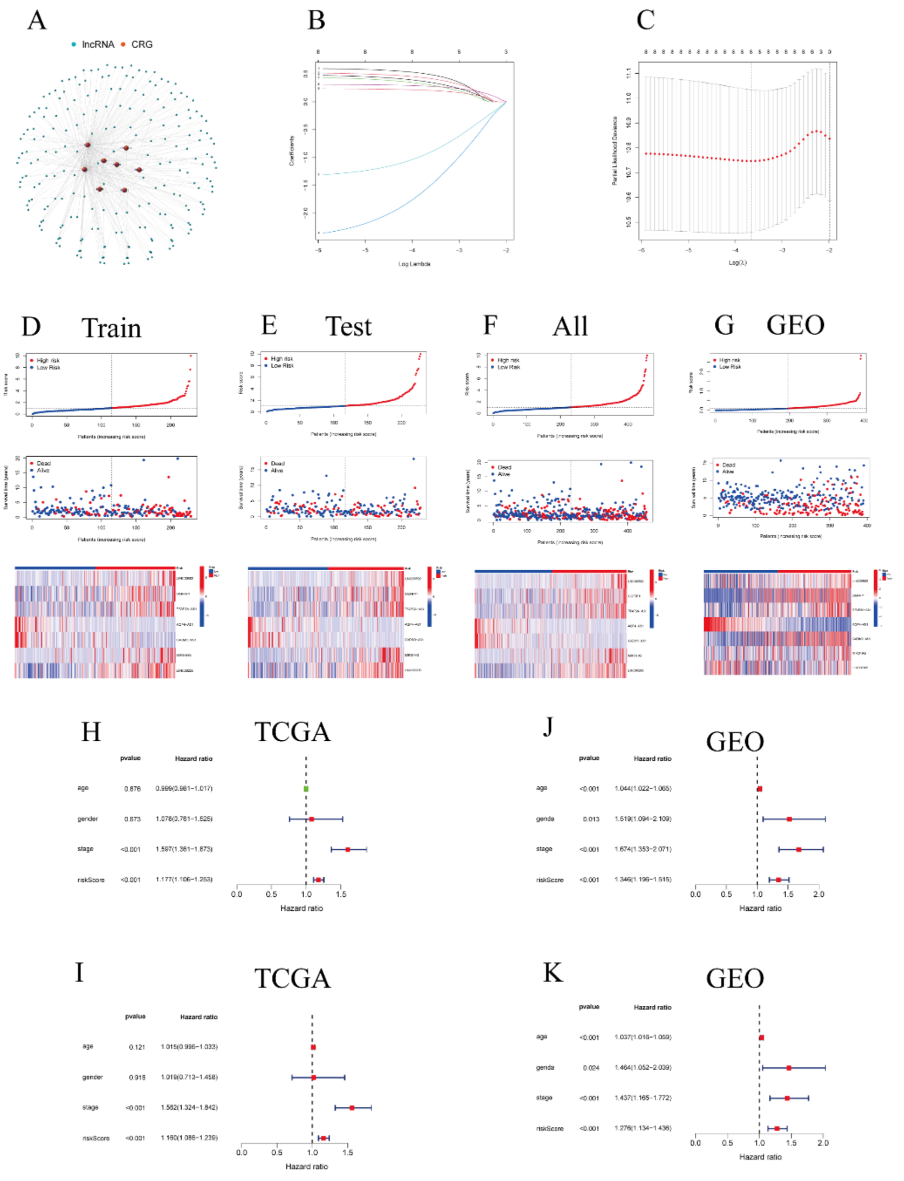

Supplementary Figure S1. Development of prognostic models (A) The correlation network diagram of 76 differentially expressed CRlncRNAs and CRGs. (B, C) The model genes were screened by LASSO regression. (D-G) The distribution of risk scores, the survival time and survival status, and the related expression of 7 CRlncRNAs between the high- and low-risk groups in the training group, the testing group, and all groups in TCGA, and the GEO samples. (D) Univariate and multivariate CRA confirmed that our risk model was an independent prognostic indicator in TCGA and GEO cohorts.

2 Supplementary Figure S2

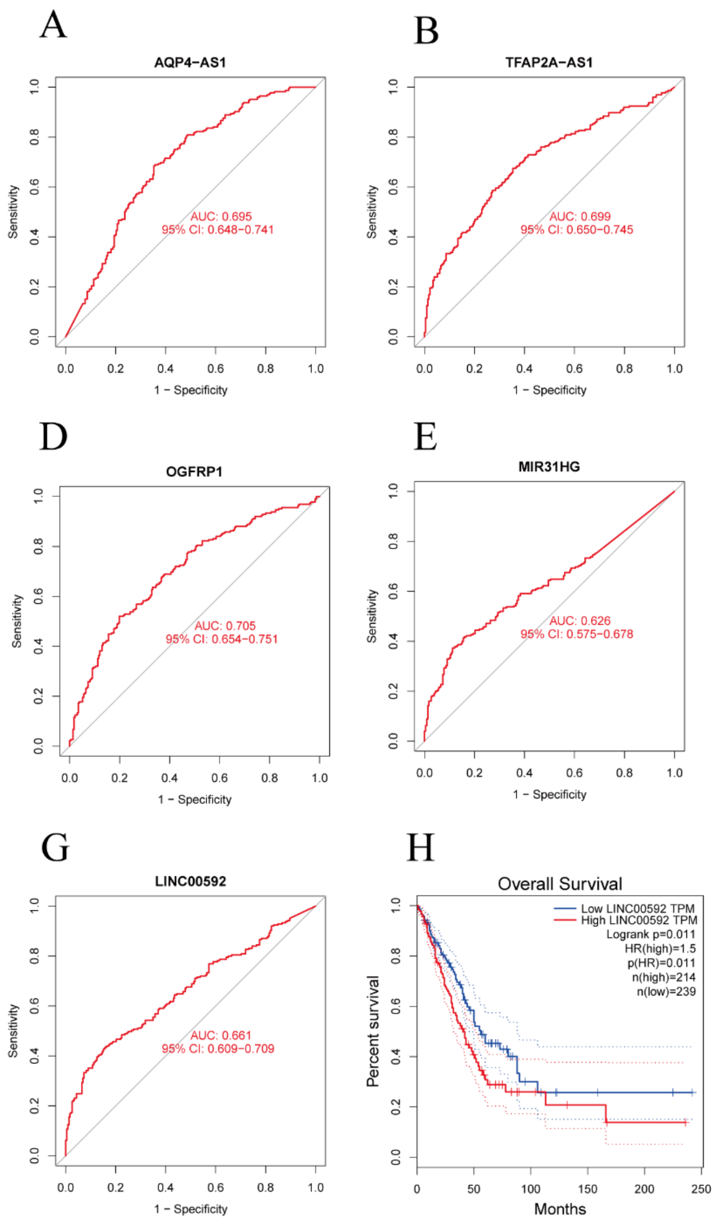

Supplementary Figure S2. Development of prognostic models (A-G) ROC curve of model genes predicting high-and low-risk patients. (H) Using the GRPIA database, a KM survival curve explains the effect of lnc on the survival of LUAD patients.
